# Supplementary material for: Does the Use of the “Proseek® Multiplex Inflammation I Panel” Demonstrate a Difference in Local and Systemic Immune Responses in Endometriosis Patients with or without Deep-Infiltrating Lesions?
Source: Int J Mol Sci. 2023 Mar 6;24(5):5022. doi: 10.3390/ijms24055022 (PMC10003683; doi:10.3390/ijms24055022)
Supplement: Supplementary file 1 [file ijms-24-05022-s001.zip › ijms-2151287-supplementary.pdf]

Table S1. List of all proteins included in the "Proseek® Multiplex Inflammation I Panel".

**Target proteins**	
Adenosine Deaminase (ADA)	Interleukin-18 receptor 1 (IL-18R1)
**Artemin (ARTN)**	**Interleukin-20 (IL-20)**
Axin-1 (AXIN1)	**Interleukin-20 receptor subunit alpha (IL-20RA)**
Beta-nerve growth factor (Beta-NGF)	**Interleukin-22 receptor subunit alpha-1 (IL-22 RA1)**
Caspase 8 (CASP-8)	Interleukin-17A (IL-17A)
C-C motif chemokine 4 (CCL4)	Interleukin-17C (IL-17C)
C-C motif chemokine 19 (CCL19)	**Interleukin-24 (IL-24)**
C-C motif chemokine 20 (CCL20)	**Interleukin-33 (IL-33)**
C-C motif chemokine 23 (CCL23)	Latency-associated peptide transforming growth factor beta 1
C-C motif chemokine 25 (CCL25)	Leukemia inhibitory factor receptor (LIF-R)
C-C motif chemokine 28 (CCL28)	**Leukemia inhibitory factor (LIF)**
CD40L receptor (CD40)	Macrophage colony-stimulating factor 1 (CSF-1)
CUB domain-containing protein 1 (CDCP1)	Macrophage inflammatory protein 1-alpha (CCL3)
C-X-C motif chemokine 1 (CXCL1)	Matrix metalloproteinase-1 (MMP-1)
C-X-C motif chemokine 5 (CXCL5)	Matrix metalloproteinase-10 (MMP-10)
C-X-C motif chemokine 6 (CXCL6)	Monocyte chemotactic protein 1 (MCP-1)
C-X-C motif chemokine 9 (CXCL9)	Monocyte chemotactic protein 2 (MCP-2)
C-X-C motif chemokine 10 (CXCL10)	Monocyte chemotactic protein 3 (MCP-3)
C-X-C motif chemokine 11 (CXCL11)	Monocyte chemotactic protein 4 (MCP-4)
Cystatin D (CST5)	Natural killer cell receptor 2B4 (CD244)
Delta and Notch-like epidermal growth factor- related recep (DNER)	Neurotrophin-3 (NT-3)
Eotaxin-1 (CCL11)	**Neurturin (NRTN)**
Eukaryotic translation initiation factor 4E-binding protein 1 (4E-BP1)	Oncostatin-M (OSM)
Fibroblast growth factor 5 (FGF-5)	Osteoprotegerin (OPG)
Fibroblast growth factor 19 (FGF-19)	Programmed cell death 1 ligand 1 (PD-L1)
Fibroblast growth factor 21 (FGF-21)	Protein S100-A12 (EN-RAGE)
Fibroblast growth factor 23 (FGF-23)	Signaling lymphocytic activation molecule (SLAMF1)
Fms-related tyrosine kinase 3 ligand (Flt3L)	SIR2-like protein 2 (SIRT2)
Fractalkine (CX3CL1)	STAM-binding protein (STAMPB)
Glial cell line-derived neurotrophic factor (GDNF)	Stem cell factor (SCF)
Hepatocyte growth factor (HGF)	Sulfotransferase 1A1 (ST1A1)
Interferon gamma (IFN-gamma)	T-cell surface glycoprotein CD5 (CD5)
**Interleukin-1 alpha (IL-1 alpha)**	T-cell surface glycoprotein CD6 isoform (CD6)
**Interleukin-2 (IL-2)**	T-cell surface glycoprotein CD8 alpha chain (CD8A)
**Interleukin-2 receptor subunit beta (IL-2RB)**	**Thymic stromal lymphopoietin (TSLP)**
**Interleukin-4 (IL-4)**	TNF-beta (TNFB)
**Interleukin-5 (IL-5)**	TNF-related activation-induced cytokine (TRANCE)
Interleukin-6 (IL-6)	TNF-related apoptosis-inducing ligand (TRAIL)
Interleukin-7 (IL-7)	Transforming growth factor alpha (TGF-alpha)
Interleukin-8 (IL-8)	Tumor necrosis factor (Ligand) superfamily, member 12 (TWEAK)
Interleukin-10 (IL-10)	**Tumor necrosis factor (TNF)**
Interleukin-10 receptor subunit alpha (IL-10RA)	Tumor necrosis factor ligand superfamily member 14 (TNFSF14)
Interleukin-10 receptor subunit beta (IL-10RB)	Tumor necrosis factor receptor superfamily member 9 (TNFRSF9)
Interleukin-12 subunit beta (IL-12B)	Urokinase-type plasminogen activator (uPA)
**Interleukin-13 (IL-13)**	Vascular endothelial growth factor A (VEGF-A)
Interleukin-15 receptor subunit alpha (IL-15RA)	
Interleukin-18 (IL-18)	

Table legend: The proteins that were excluded from further analysis in plasma due to their expression in less than 50% of patients are set in bold, those excluded in PF are underlined.
